# Nephrotic Syndrome Associated with Lung Cancer: A Rare Case of Malignancy Associated with AA Amyloidosis

**DOI:** 10.1155/2013/831903

**Published:** 2013-03-28

**Authors:** Victor Gueutin, Anne-Lyse Langlois, Nathalie Shehwaro, Ryme Elharraqui, Philippe Rouvier, Hassane Izzedine

**Affiliations:** ^1^Department of Nephrology, Pitié-Salpêtrière Hospital, Assistance Publique-Hôpitaux de Paris, Université Paris VI Pierre et Marie Curie, 47-83 Boulevard de l'Hôpital, 75013 Paris, France; ^2^Department of Pathology, Pitié-Salpêtrière Hospital, Assistance Publique-Hôpitaux de Paris, Université Paris VI Pierre et Marie Curie, 47-83 Boulevard de l'Hôpital, 75013 Paris, France

## Abstract

Nonhematologic malignancies are rarely reported to be associated with AA amyloidosis. Although the association between renal cell carcinoma and systemic AA amyloidosis has been established, the evidence linking pulmonary cancer to AA amyloidosis is scarce. Here, a case of biopsy-proven renal AA amyloidosis complicated with nephrotic syndrome associated with lung carcinoma is reported.

## 1. Introduction

Secondary (AA) amyloidosis is a disorder characterised by the extracellular tissue deposition of fibrils that are composed of fragments of serum amyloid A (SAA) protein [1]. Chronic inflammatory disease is a major cause of AA amyloidosis and nonhematologic malignancies are rarely reported to be associated with AA amyloidosis [2]. Excluding the tumors associated with localized amyloid, the incidence of generalized amyloidosis in patients with cancer has been estimated to be between 0.1 and 0.4% among all cancers [3]. From all cancers, renal carcinoma appears to be an important exception, because these tumors are responsible for 25–33% of all cancers associated with amyloidosis [4]. This low incidence of malignancy-related systemic AA amyloidosis seems to be linked to the short-term survival of cancer patients who died before significant systemic deposition of amyloid fibrils can occur. Although the association between renal cell carcinoma and systemic AA amyloidosis has been established, the evidence linking pulmonary cancer to AA amyloidosis is scarce [2, 5–7] and few reports concern mainly non small cell lung cancer and AA amyloidosis manifested by nephrotic syndrome [2, 5–8]. We report here the case of nephrotic syndrome and renal failure due to systemic AA amyloidosis in a patient with nonsmall cell lung carcinoma (NSCLC).

## 2. Case Report

A 56-year-old man was referred to our hospital because of generalized edema, renal failure, and proteinuria. Chemounresponsive advanced NSCLC (stage IIIB adenocarcinoma) had been diagnosed on the left upper lung one year earlier. Medical history was unremarkable for recurrent infectious or chronic inflammatory disease, familial hypotension or neuropathy, and familial Mediterranean fever. Biochemical screening of renal function had been normal two months previously (baseline serum creatinine level of 0.76 mg/dL). 

Physical examination findings revealed generalized edema without breathing sound on the upper zone of the left lung. His blood pressure was 80/60 mmHg. Urinalysis revealed 3+ proteinuria. Laboratory data were as follows: white blood cell count was 28,100/mm^3^, red blood cell count was 250 × 10^4^/*μ*L, hemoglobin was 10.8 g/dL, hematocrit was 36.1%, platelet count was 20.8 × 10^4^/mm^3^, total serum protein was 6.4 g/dL, albumin was 1.25 g/dL, creatinine was 3.09 mg/dL, sodium was 137 mEq/L, potassium was 4.0 mEq/L, and chloride was 102 mEq/L. The erythrocyte sedimentation rate (ESR) (120 mm/h) and C-reactive protein (CRP) (6.93 mg/dL) were elevated, but no infections were found. The urine was 4+ for protein and 10–15 red blood cells were observed per high power field. Twenty-four-hour urinary protein excretion was 12 g; Bence-Jones proteinuria was not present. Serum protein and immunoelectrophoresis revealed hypoalbuminaemia with no monoclonal gammopathy rheumatoid factor, hepatitis B and C antigens, HIV serology, anti-GBM antibodies, myeloperoxidase and proteinase 3-antineutrophil cytoplasmic antibodies, and antinuclear antibody were all negative as well as tuberculin skin test. Ultrasound cardiography demonstrated good wall movement with a 58% ejection fraction, normal diastolic function, and mild aortic and pulmonary regurgitation and without granular pattern due to amyloid deposition. 

**Figure 1:**
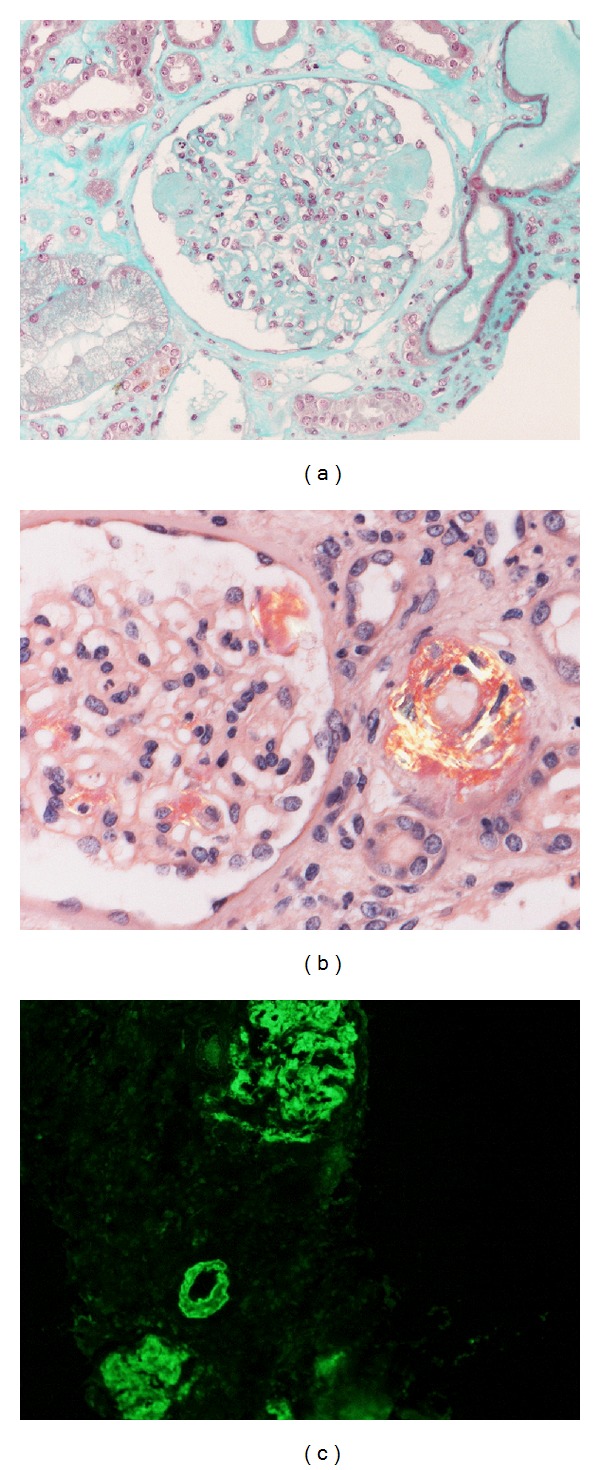
Histopathological findings of kidneys. (a) nodular appearance due to amorphous acellular mesangial deposition (Masson trichrome stain; magnification, ×200). (b) accumulation of amyloid expanding the mesangial area and the arteriole (Congo red stain viewed by polarized light microscopy), (c) immunohistochemically positivity of AA amyloid (avidin-biotin-peroxidase complex system; original magnification, ×200).

Transjugular kidney biopsy was performed and histological examination showed homogenous massive amyloid deposits in glomeruli (Figure 1(a)) and vessel walls. This material produced apple-green birefringence under polarized light, (Figure 1(b)), by Congo red staining. The amyloid stained strongly with anti-AA (Figure 1(c)) but not with anti-*κ* or anti-*λ* antiserum. Renal function deteriorated rapidly and the patient underwent palliative treatment.

## 3. Discussion

In our case, the absence of classic causes of chronic inflammation or infection suggests that AA amyloidosis was induced by the lung carcinoma.

In clinical series, the prevalence of renal involvement in patients with cancer is rather oriented glomerulopathies other than amyloidosis (e.g., membranous nephropathy, IgA nephropathy, minimal change nephropathy, rapidly progressive GN, membranoproliferative GN, crescentic glomerulonephritis, etc.). Lee et al. found that 11% of patients with the nephrotic syndrome had carcinoma [9].

Lung cancer is the leading cause of cancer deaths worldwide. The Tromso study describing an association between albuminuria and cancer showed a 5.4-fold increase risk for lung cancers in patients with an albumin-to-creatinine ratio in the highest quintile [10]. Lung cancer is among the malignancies commonly associated with a paraneoplastic nephrotic syndrome mostly related to membranous nephropathy [11]. The incidence of secondary AA amyloidosis during lung cancer is unknown. In a necropsy series, amyloid occurred in 6–10% of bronchogenic carcinomas [12]; unfortunately, histological type was not mentioned. In 310 cases of squamous cell carcinoma with distal obstructive pneumonitis, no evidence of amyloidosis in the adjacent lung was found [6]. Furthermore, from 24,388 consecutive autopsies revealing 148 cases of amyloidosis, no cases of lung cancer were noted [13]. Indeed, only few cases of lung cancer presenting as renal AA amyloidosis have been reported (Table 1) [2, 5–9].

Renal AA amyloidosis seems to occur in the early years of evolution of lung cancer disease, mostly in nonsquamous cell lung cancer type. Recent data suggests that 20% of patients with metastatic renal cell carcinoma and high serum albumin A (SAA) levels survive for 5 years or more before developing systemic AA amyloidosis [14]. Renal abnormalities included nephrotic syndrome and acute or chronic renal failure that underwent dialysis.

In cancer patients, AA amyloidosis may be caused by the overproduction of SAA. We are not aware of the SAA level of our patient, but his CRP concentration had been elevated for at least 1 year prior to the current medical problem. Serum C-reactive protein and SAA correlate highly [15], and given the association between malignancies and serum amyloid A concentrations, a plausible hypothesis for the origin of our patient's AA amyloidosis would be a high SAA titre produced by the NSCLC. In the case of pulmonary metastasis of renal cancer [2] histopathological studies revealed that the tumor cells were surrounded by numerous lymphocytes, macrophages, and plasma cells, and AA amyloid was deposited in the outer layer of tumor cells and reactive cells, which suggests that the malignant cells were being encapsulated by immune cells. Indeed, proinflammatory cytokines (IL-1*β*, IL-6, and TNF-*α*) produced by antitumor lymphocytes or macrophages can induce SAA production and development of AA amyloidosis. 

In conclusion, lung cancer could be included in the list of neoplastic diseases predisposing to AA amyloidosis. 

## Figures and Tables

**Table 1:** Biopsy-proven renal AA Amyloidosis associated with lung cancer.

Author, year	Age (year)/sex	Cancer type and stage	Occurrence of renal damage in relation to cancer diagnosis	Renal presentation	Treatment	Outcome
Meyrier et al. [5] 1985	59/M	NSCLC	One year ago	NS	Chemotherapy	Died
Richmond et al. [6] 1990	72/M	Bronchial carcinoma	Simultaneously	Necropsic diagnosis	None	Died
Garthwaite et al. [7] 2003	64/M	Bronchial SCC	Simultaneously	NS, RI	Palliative	Hemodialysis
Paydas et al. [8] 2005	50/M	NSCLC IIIB	2 years ago	NS, CRF	Patient refusal	Hemodialysis
Nobata et al. [2] 2012	71/M	Metastatic lung tumor from RCC	RCC 8 years ago	NS, ARF on CKD	Surgery	Hemodialysis
Our case	56/M	NSCLC IIIB	1 year ago	NS, ARF	Palliative	Renal function worsening

M: male, SCC: squamous cell carcinoma, NS: nephrotic syndrome, RI: renal insufficiency, CRF: chronic renal failure, ARF: acute renal failure, NSCLC: non small cell lung cancer, and RCC: renal cell carcinoma.
